# Modulation of benzo[a]pyrene–DNA adduct formation by CYP1 inducer and inhibitor

**DOI:** 10.1186/s41021-017-0076-x

**Published:** 2017-04-10

**Authors:** Kazuhiro Shiizaki, Masanobu Kawanishi, Takashi Yagi

**Affiliations:** 1 Department of Applied Biosciences, Faculty of Life Sciences, Toyo University, Itakura, Gunma 374-0193 Japan; 2 Department of Biological Science, Graduate School of Science, Osaka Prefecture University, Sakai, Osaka Japan

**Keywords:** DNA adduct, Benzo[*a*]pyrene, Aryl hydrocarbon receptor, Cytochrome P450

## Abstract

Benzo[*a*]pyrene (BaP) is a well-studied pro-carcinogen that is metabolically activated by cytochrome P450 enzymes. Cytochrome P4501A1 (CYP1A1) has been considered to play a central role in the activation step, which is essential for the formation of DNA adducts. This enzyme is strongly induced by many different chemical agents, including 2,3,7,8-tetrachlorodibenzo-*p*-dioxin (TCDD), which binds to the aryl hydrocarbon receptor (AhR). Therefore, AhR activators are suspected to have the potential to aggravate the toxicity of BaP through the induction of CYP1A1. Besides, CYP1A1 inhibitors, including its substrates, are estimated to have preventive effects against BaP toxicity. However, strangely, increased hepatic BaP–DNA adduct levels have been reported in *Cyp1a1* knockout mice. Moreover, numerous reports describe that concomitant treatment of AhR activators reduced BaP–DNA adduct formation. In an experiment using several human cell lines, TCDD had diverse modulatory effects on BaP–DNA adducts, both enhancing and inhibiting their formation. In this review, we focus on the factors that could influence the BaP–DNA adduct formation. To interpret these complicated outcomes, we propose a hypothesis that CYP1A1 is a key enzyme for both generation and reduction of (±)-anti-benzo[a]pyrene-7,8-diol-9,10-epoxide (BPDE), the major carcinogenic intermediate of BaP. Conversely, CYP1B1 is thought to contribute only to the metabolic activation of BaP related to carcinogenesis.

## Background

Benzo[*a*]pyrene (BaP) is a well-researched precarcinogen found in cigarette smoke, cooked food, and various combustion gases, such as vehicle exhausts [1–3]. BaP is biotransformed by several enzymatic steps to its ultimate carcinogenic forms, which are highly reactive electrophilic intermediates and form DNA adducts by covalent binding [1, 4–6] The ultimate carcinogenic intermediate of BaP is (±)-anti-benzo[*a*]pyrene-7,8-diol-9,10-epoxide (BPDE). The metabolic enzymes required for BPDE production are thought to be cytochrome P4501A1 and 1B1(CYP1A1 and 1B1). These enzymes are highly inducible by polycyclic aromatic hydrocarbons (PAHs), such as 3-methylcholanthrene and BaP [7]. This induction is directed by a ligand-activated transcriptional factor named aryl hydrocarbon receptor (AhR) [8, 9], known as a dioxin receptor. Following the binding to ligands, AhR translocates to the nucleus and forms a heterodimer with AhR nuclear translocator (ARNT) [10]. The AhR/ARNT complex enhances the transcription of target genes including CYP1A1, 1A2, and 1B1 [11]. Therefore, BaP enhances its own metabolic enzyme expression by binding to AhR as a ligand and acquiring mutagenicity [12]. Structural diversity of exogenous and endogenous AhR ligands has been reported [13]. They are found not only in combustion products but also in food, such as dietary herbal supplements, vegetables, and fruits. These ligands would be ingested daily and could aggravate the toxicity of BaP through the induction of metabolic enzymes [14]. In contrast, AhR antagonists, which can prevent CYP enzyme induction, have the possibility of reducing BaP adducts [15–17]. This indicates the possibility of modulating BaP toxicity by altering AhR activity. In fact, lack of BaP carcinogenicity in the skin of AhR knockout mice was observed [18]. In addition, AhR agonists would be expected to aggravate BaP toxicity through CYP enzyme induction, while AhR antagonists may prevent such toxicity. Contrary to this assumption, in vivo and in vitro experiments have shown that BaP–DNA adduct formation was suppressed by pretreatment with a potent AhR agonist, 2,3,7,8-tetrachlorodibenzo-*p*-dioxin (TCDD) [19–21]. Strangely, AhR knockout mice exhibited an enhanced BaP adduct level after the oral administration of BaP [22]. Other studies on the effects of AhR activator on BaP–DNA adduct formation also appeared to provide paradoxical results [23–29]. Table 1 shows a summary of previous studies about the alteration of BaP toxicity and adduct formation by natural compounds and artificial chemicals. Because these compounds include not only AhR agonist ligands but also non-ligand AhR activator and antagonist ligands, we refer to them as AhR modulators in this review. Many AhR modulators are substrates of CYP enzymes and some of them are known as CYP inhibitors [30]. This might be one of the reasons why these various and complicated results have been obtained.

**Table 1:** Influence of benzo[a]pyrene DNA adduct by various compounds

Reference	Cells/organs	Compound	Physiological property	Modulation of BaP adduct
de Waard et al. [20]	Caco-2 (human colorectal carcinoma)	TCDD	AhR agonist	reduction
Gelhaus SL [21]	H358 (human lung carcinoma)	TCDD	AhR agonist	enhancement
Wu Q et al. [19]	mouse liver	TCDD	AhR agonist	reduction
Harrigan JA et al. [23]	rat lung and liver (ex vivo)	TCDD	AhR agonist	enhancement
Shiizaki K et al. [24]	HepG2 (human hepatoma)	TCDD	AhR agonist	reduction
Hodek P et al. [15]	rat liver/small intestine	BNF	AhR agonist	enhancement
Chang KW et al. [25]	A427 and CL3 (human lung carcinoma)	BNF ANF	AhR agonist AhR antagonist/CYP1A1 inhibitor	enhancement reduction
Mohebati A et al. [16]	MSK-Leuk	ANF carnosol	AhR antagonist/CYP1A1 inhibitor (AhR antagonist)	reduction reduction
Takemura H et al. [26]	MCF-7 (human breast adenocarcinoma)	chrysoeriol	CYP1A1/1B1 inhibitor	reduction
Wen X et al. [27]	SCC-9 (human squamous carcinoma)	5,7-DMF	CYP1B1 inhibitor	reduction
Kang ZC et al. [14]	HepG2	quercetin	AhR activator	reduction
Vanhees K et al. [54]	mouse liver (ex vivo)	quercetin	AhR activator	reduction
Revel A et al. [17]	mouse lung	resveratrol	AhR antagonist	reduction
Sehgal A et al. [28]	mouse liver and lung	curcumin	CYP1A1 inhibitor/(AhR agonist)	reduction
Kleiner HE et al. [29]	MCF-7 (human breast adenocarcinoma)	coumarins	CYP1A1/1B1 inhibitor	reduction

*BNF* β-naphthoflavone, *ANF* α-naphthoflavone, *5,7-DMF* 5,7-dimethoxyflavone

After summarizing the enzymes involved in BaP metabolism, we provide a range of experimental results about the effects of AhR modulators on BaP adduct formation. Finally, this review focuses on the expression profile of CYP isoforms in cells in order to interpret these complicated, paradoxical, and enigmatic results.

## BaP catalytic enzymes and their induction by AhR

Several mammalian enzymes involved in BaP metabolism have been reported [31–34]. CYP1A1, 1A2, 1B1, 2C9, 2C19, and 3A4 are considered to be the oxidation enzymes of BaP. Among these enzymes, BaP metabolites that can form DNA adducts were generated by CYP1A1, 1B1, and 2C19 [24]. The products generated by these CYP subtypes are considered to be benzo[a]pyrene 7,8-epoxide and BPDE. CYP1A1 is expressed ubiquitously and CYP2C19 is expressed specifically in the liver, while CYP1B1 is expressed in extrahepatic tissues [35, 36]. AhR regulates the inducible expression of the CYP1A1 and 1B1 genes, but not that of CYP2C19 [37]. Therefore, the expression of these two enzymes would contribute to the modulation of BaP–DNA adduct formation in the presence of additional AhR ligands other than BaP. Another enzyme involved in BaP metabolism is epoxide hydrolase (EPHX). Benzo[a]pyrene-7,8-epoxide, an CYP1A1 or CYP1B1 metabolite of BaP, is transformed to benzo[a]pyrene-7,8-dihydrodiol by microsomal epoxide hydrolase (EPHX1). Then, benzo[a]pyrene-7,8-dihydrodiol is catalyzed to BPDE by CYP1A1 or CYP1B1. EPHX1 and CYP are considered to generate BPDE in a coordinated manner [38]. EPHX1 gene regulation by the transcription factor GATA-4 has been reported, but EPHX1 is not an AhR target gene [24, 39]. For the conjugating enzymes, UDP-glucuronosyltransferase (UGT) subtypes UGT1A1 and UGT1A6 and glutathione transferase (GST) subtypes GSTA1, GSTA2, GSTA4, GSTM1, GSTP1, and microsomal GST play roles in generating hydrophilic conjugates. Among these enzymes, UGT1A1 induced by TCDD via AhR was reported. Overall, CYP1A1, CYP1B1, and UGT1A1 are most likely to influence BaP–DNA adduct formation via AhR activators.

## Suppression of BaP adduct formation by TCDD

**Fig. 1:**
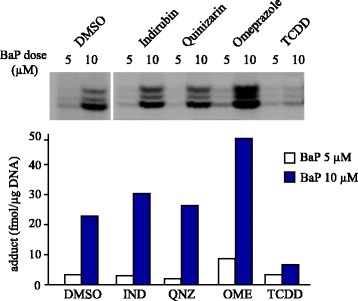
Alteration of BaP–DNA adduct formation by CYP subtypes and AhR activators. Effect of AhR activator on BaP–DNA adduct formation in HepG2 cells. HepG2 cells were concomitantly treated with BaP (5 or 10 μM), indirubin (IND, 100 nM), quinizarin (QNZ, 10 μM), omeprazole (OME, 100 μM), or TCDD (10 nM) for 16 h. After incubation, DNA was purified and BaP–DNA adducts were analyzed. This figure is adapted from data reported previously [24]

To investigate the modulation of BaP-induced DNA adduct formation by AhR agonists, TCDD is considered to be the most favorable ligand. This is because TCDD has been identified as the most potent ligand of AhR and shown not to be biotransformed and could not form any DNA adducts. An in vivo study showed that treatment with TCDD prior to BaP exposure suppressed the formation of BaP-induced DNA adducts in mouse liver. In human colon carcinoma cells Caco2 and human lung carcinoma cells H358, BaP–DNA adduct formation was found to be suppressed by TCDD treatment [20, 21]. In our study, concomitant exposure to AhR activators and BaP showed a wide variety of both protective and aggravative effects. Figure 1 shows the alteration of BaP adduct formation by various AhR ligands and activators in human hepatoma HepG2 cells (adapted from data reported previously [24]). Remarkably, TCDD significantly reduced BaP-induced mutagenicity and the protective effects. These protective effects by TCDD were more significant upon BPDE exposure and were estimated to involve induction of a BPDE catalytic enzyme [24]. Among candidate enzymes for this, UGT1A1 was considered to be the most likely because it is natural that conjugating enzymes should reduce the activated form of BaP; however, in this report, sulforaphane-induced UGT1A1 did not suppress any DNA adduct formation. Besides, CYP1A1 artificially induced due to the “tet-on gene regulation system” reduced BaP–DNA adduct formation induced by BPDE [24]. Therefore, CYP1A1 is the most likely candidate for an enzyme that can transform BPDE to the non-adduct-forming metabolites among the TCDD-inducible drug-metabolizing enzymes.

## Alteration of BaP adduct formation by CYP substrates and inhibitors

AhR modulators showed a wide variety of both protective and aggravative effects on BaP adduct formation, as shown in Fig. 1. Indirubin, one of the endogenous AhR ligands [40], showed a slight preventive effect. However, the phyto-anthraquinone alizarin [41], which is also regarded as an AhR agonist, did not show any preventive effects. These results can be interpreted as follows. Indirubin and alizarin are not only AhR agonists but also CYP1A1 substrates, which could represent competitive inhibition. From the hypothesis that CYP1A1 activity is crucial for BaP–DNA adduct formation, these chemicals would have dual functions for CYP1A1 and not simply act like TCDD. On the other hand, BaP–DNA adducts were increased by concomitant exposure to BaP and omeprazole. Omeprazole, a drug for treating gastro-esophageal reflux disease, activates AhR without binding as a ligand [42, 43]. Omeprazole-mediated CYP1A1 induction requires more than 12 h after omeprazole treatment and it is slower than the BaP-mediated CYP1A1 induction [44]. Moreover, omeprazole has inhibitory effects on CYP1A1 as a competitive inhibitor [45]. Thus, the increase of BaP adducts by omeprazole would be a result that reflected only CYP1A1 inhibition, but not CYP1A1 induction. An AhR antagonist, α-naphthoflavone, also increased BaP adduct formation, and the enhancing effects would be due to CYP1A1 inhibition rather than the effects as an AhR antagonist. In our previous studies, BPDE-induced DNA adducts were reduced by recombinant CYP1A1, and additional α-naphthoflavone induced recovery from this reduction [24]. This in vitro assay system was unrelated to AhR-mediated gene transcriptional regulation, so the results support the hypothesis that CYP1A1 can metabolically reduce BPDE. The enhancement of BaP–DNA adducts by CYP1A1 inhibitors including substrates would be the result of inhibition of the metabolic elimination of BPDE.

## Expression of CYP isoforms and BaP–DNA adduct formation

**Fig. 2:**
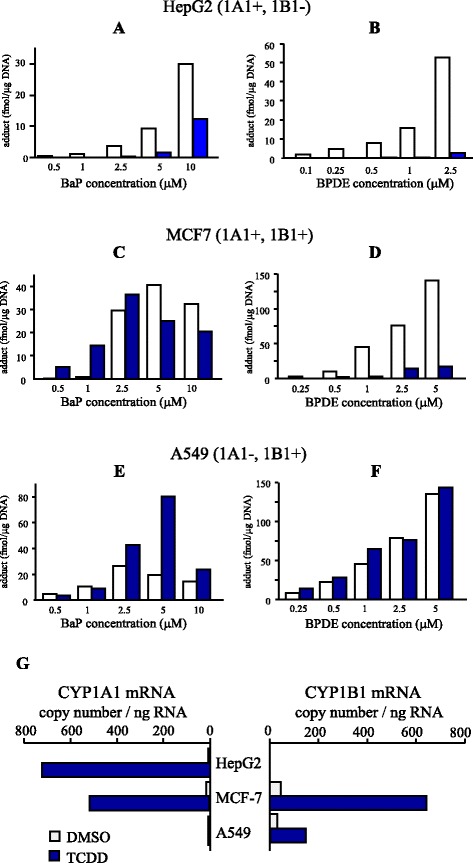
TCDD-mediated modulation of BaP adducts in various human cell lines. Hepatoma cell line HepG2 (**a**, **b**), breast carcinoma cell line MCF7 (**c**, **d**), and lung carcinoma cell line A549 (**e**, **f**) were co-treated with BaP (0.5–10 μM; **a**, **c**, **e**) or BPDE (0.1–5 μM; **b**, **d**, **f**) with or without TCDD. Closed columns represent adducts by 10 nM TCDD treatment and open columns represent adducts by solvent control (DMSO). After 16 h of incubation, genomic DNA was isolated and DNA adducts were detected by ^32^P-postlabeling and PAGE. **G**: CYP1A1 and 1B1 mRNA expression in the three cell lines. Each cell line was exposed to 10 nM TCDD or DMSO for 24 h and total RNA was extracted. For the quantitation of specific transcripts, real-time PCR was performed. These figures are adapted from data reported previously [24]

The induction of CYP1A1 by an AhR agonist caused a reduction of BaP–DNA adducts in HepG2 cells. However, this cell line lacks the expression of CYP1B1, which is another AhR-inducible CYP enzyme transforming BaP to BPDE. The importance of CYP1B1 in BaP metabolism has been well studied both in vivo and in vitro, as has that of CYP1A1 [46–48]. *Cyp1a1* knockout mice have shown higher levels of BaP–DNA adducts [38]. However, the loss of Cyp1b1 had little impact on tumor response to BaP [22, 48]. In ex vivo experiments, CYP1B1 polymorphism and BaP–DNA adducts were also shown to be well correlated [49]. Figure 2 shows the results of our in vitro study using several human cell lines, which express different patterns of CYP isoforms (adapted from data reported elsewhere [24]). The modulation of BaP–DNA adducts by TCDD was well correlated to CYP1A1 and CYP1B1 expression. TCDD enhanced BaP–DNA adduct formation in A549 cells that express CYP1B1 but not CYP1A1. Furthermore, TCDD did not influence BPDE-induced DNA adduct formation. In MCF-7 cells expressing both CYP1A1 and CYP1B1, the modulation by TCDD exhibited dual features. TCDD induced adduct formation upon exposure to a low dose of BaP. However, it reduced adduct formation upon high-dose BaP exposure. In addition, BPDE-induced adduct formation was reduced by TCDD, similar to the results observed with HepG2 cells. These findings can be interpreted as follows: CYP1A1 would be a key enzyme for both the generation and the reduction of BPDE, the major carcinogenic intermediate of BaP, while CYP1B1 has only the former activity. Therefore, it is estimated that various outcomes of changes in adduct formation by concomitant exposure to BaP and AhR agonist would arise from the cellular expression of CYP1 isoforms. Similar results were obtained in a study by Genies et al. comparing BaP toxicity in HepG2 and A549 cells; they emphasized the large differences in the responses of cells originating from different organs [50].

## Discussion

**Fig. 3:**
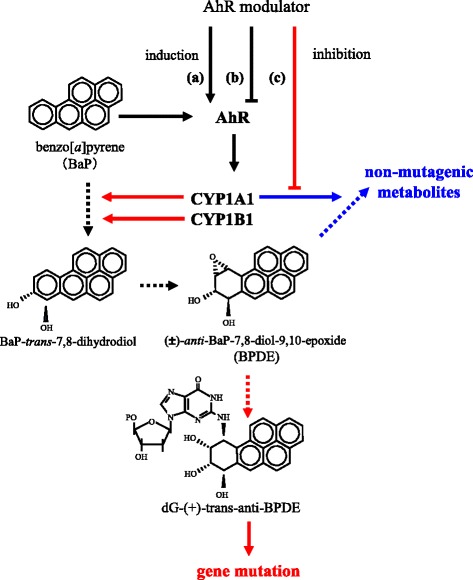
Scheme of the relationship between AhR modulators and BaP–DNA adduct formation. *Broken arrows* indicate the pathway of BaP metabolism. *Solid arrows* indicate the activation or inductive commitment, whereas the T-shaped lines indicate inhibition. The “protective pathway” and “aggravating pathway” are indicated in *blue* and *red*, respectively. AhR modulators influence BaP adducts through CYP1A1/1B1 gene induction (a), reduction (b), and/or inhibition of enzyme activity (c). Note that only CYP1A1 has a BPDE metabolic detoxification indicated by a *blue arrow*

Numerous studies have reported on the effects of concomitant exposure or pretreatment of AhR ligands on BaP toxicity. As shown in Table 1, diverse effects, namely, both enhancement and reduction of BaP–DNA adduct formation, were identified. We assert that one of the causes of the earlier confusion is the dual functions of the CYP1A1 enzyme against BaP metabolism. Studies focusing on CYP1A1 regarding BaP toxicity have mainly involved its carcinogenicity via BPDE production over a long period. However, a recent study revealed that CYP1A1 has detoxifying activity on BaP [22]. Our experimental results also constitute evidence that CYP1A1 mediates the elimination of BPDE, and it was estimated that the AhR agonist can enhance the generation and elimination of BPDE via the induction of CYP1A1. Furthermore, the modulation of BaP adduct formation by AhR ligand would not be simple because the compound itself occasionally affects CYP enzymes as well as ligands. For example, the widely used AhR antagonist α-naphthoflavone is an inhibitor of CYP1A1 at the same time, so it can inhibit not only CYP1A1 gene transcription but also enzyme activity. In addition, omeprazole is a CYP1A1 inhibitor as well as an AhR activator and enhanced BaP adduct formation. The enhancement by omeprazole would result from CYP1A1 inhibition rather than CYP1A1 induction via AhR. Among AhR-regulated drug-metabolizing enzymes besides CYP1A1, CYP1B1 had a substantial influence on BaP adduct formation. This enzyme seems to take part in only BPDE production in the process of BaP-induced adduct formation. The difference between CYP1A1 and CYP1B1 in BPDE metabolism impacted not only the sensitivity to BaP, but also the TCDD-mediated modulation of BaP adduct formation. The scheme in Fig. 3 indicates the relationship between AhR modulators and BaP–DNA adducts. Broken arrows indicate the pathway of BaP metabolism. Solid arrows indicate the activation or inductive commitment, whereas the T-shaped lines indicate inhibition. The “protective pathway” and “aggravating pathway” are indicated in blue and red, respectively. AhR modulators influence BaP adducts through CYP1A1/1B1 gene induction (a), reduction (b), and/or inhibition of enzyme activity (c). A recent report showed that impact of individual cytochrome P450 enzymes including CYP1A1 and 1B1 in BaP metabolism [51]. The report showed that CYP1A1 and 1B1 also generate products of detoxification such as 3-hydroxy-BaP. The role of CYP1B1 in the detoxification of BaP would be limited to the production of these non-reactive metabolites.

CYP1A1 and 1B1 exhibit various tissue distributions. For example, CYP1B1 is abundant in kidney and bone marrow, but these organs scarcely express CYP1A1. Thus, alteration by AhR agonist to the BaP-adduct formation might depend on the expression profile of CYP1A1 and CYP1B1 in the target tissue. Uno et al. reported tissue-specific differences of BaP toxicity in CYP1A1- and/or CYP1B1-deficient mice [52]. They also reported the importance of BaP detoxification by CYP1A1 [22]. In addition, Shi et al. reported the differences in the role of CYP1A1 in BaP detoxification between the small intestine and liver by generating tissue-specific knockout mouse models [53]. The insights obtained from in vitro studies can consistently explain the results of these in vivo studies.

At present, we cannot definitively conclude that AhR ligands enhance BaP toxicity in the human body. However, we can predict their effects in organ exhibiting biased expression of CYP1 enzymes, for example, predominant expression of CYP1B1 in lung, and scarce expression of CYP1B1 in liver [54, 55]. This could explain some of the organ differences in the incidence of cancer caused by smoking and may contribute to developing chemoprevention using AhR ligands or CYP inhibitors.

## Conclusion

The action of the AhR modulators on BaP adduct formation is the composite result of the several effects including induction, reduction and inhibition of the CYP1 enzymes. CYP1A1 is involved in both the generation and the degradation of BPDE, while CYP1B1 only has activity in generating BPDE. The effects of AhR modulators on BaP–DNA adduct formation depend on the CYP1A1/1B1 expression profile of cells.

## Abbreviations

AhR: Aryl hydrocarbon receptor
ARNT: AhR nuclear translocator
BaP: Benzo[*a*]pyrene
BPDE: (±)-anti-benzo[*a*]pyrene-7,8-diol-9,10-epoxide
CYP: Cytochrome P450
GST: Glutathione transferase
TCDD: 2,3,7,8-tetrachlorodibenzo-*p*-dioxin
UGT: UDP-glucuronosyltransferase
